# Assessing Pharmacists’ Use and Perception of AI Chatbots in Pharmacy Practice: Cross-Sectional Survey Study

**DOI:** 10.2196/71767

**Published:** 2025-11-21

**Authors:** Anly Li, Amy Heck Sheehan, Christopher Giuliano, Paul Dobry, Paul Walker, Jennifer Philips, Joseph Jordan

**Affiliations:** 1 Regulatory Pharmaceutical Fellow in Drug Information Purdue University College of Pharmacy West Lafayette, IN United States; 2 Professor of Pharmacy Practice Purdue University College of Pharmacy West Lafayette, IN United States; 3 Clinical Professor of Pharmacy Practice Wayne State University Eugene Applebaum College of Pharmacy and Health Sciences Detroit, MI United States; 4 Clinical Pharmacy Specialist, Drug Information Detroit Receiving Hospital Detroit, MI United States; 5 Clinical Professor Emeritus University of Michigan College of Pharmacy Ann Arbor, MI United States; 6 Clinical Professor of Pharmacy Practice, Director of Drug Information Group University of Illinois Chicago College of Pharmacy Chicago, IL United States; 7 Professor of Pharmacy Practice Butler University College of Pharmacy and Health Sciences Indianapolis, IN United States

**Keywords:** chatbot, artificial intelligence, pharmacy, health care practice, survey, technology

## Abstract

**Background:**

The use of artificial intelligence (AI)–based large language model chatbots such as ChatGPT has become increasingly popular in many disciplines. However, concerns exist regarding ethics, legal considerations, accuracy, and reproducibility with its use in health care practice, education, and research.

**Objective:**

This study aimed to assess current perceptions and use of AI chatbots in pharmacy practice from the perspective of pharmacist preceptors and determine factors that may influence the use of AI chatbots in practice.

**Methods:**

A cross-sectional survey of pharmacy practice preceptors from Indiana, Illinois, and Michigan was conducted using the validated Technology Acceptance Model Edited to Assess ChatGPT Adoption (TAME-ChatGPT) survey tool to collect information regarding current use of AI chatbots and factors associated with use, including ease of use, perceived risk, technology or social influences, anxiety, and perceived usefulness.

**Results:**

A total of 194 responses (194/1877, 10.34% response rate) were received. Approximately one-third (n=59, 30.4%) of respondents reported having used an AI chatbot, with 51.5% (n=100) indicating that they planned to start or would continue using chatbots in the future. In practice, common uses for AI chatbots included summarizing information (n=90, 46.4%), letter of recommendation writing (n=64, 32.9%), and obtaining disease state information (n=63, 32.5%). The 2 main constructs associated with the use of chatbots identified from the TAME-ChatGPT tool included perceived risk of using AI and attitude toward AI. Factors that predicted pharmacists’ current use of AI chatbots included positive attitude toward technology (odds ratio [OR] 3.64, 95% CI 2.08-6.36), coworker use of AI (OR 7.41, 95% CI 2.64-20.8), and working in academia (OR 5.62, 95% CI 1.30-24.23).

**Conclusions:**

Most pharmacist respondents had not used an AI chatbot and were unlikely to make patient care decisions based on information from a chatbot. The TAME-ChatGPT survey is validated for assessing chatbot use and attitudes among pharmacists, and future studies using this survey tool can guide the implementation of chatbots into pharmacy practice.

## Introduction

### Artificial Intelligence Chatbots

In November 2022, the artificial intelligence (AI)–based large language model chatbot ChatGPT was launched [1]. It quickly gained popularity, amassing over 100 million monthly users in just 2 months [2]. Since the release of ChatGPT, several other AI chatbots have debuted, including Gemini, Microsoft Bing AI, and Copilot. This expansion in AI chatbots has led to the diversification of their use in fields outside of the technology industry. ChatGPT has been explored for use in health care practice, education, and research, and specialized chatbots such as OpenEvidence and Dougall GPT have emerged, catering specifically to clinicians and health care workers [3-6].

### Use of Artificial Intelligence in Healthcare

A survey evaluating the use and perceptions of ChatGPT in health care professionals was conducted at Northwestern University [7]. In addition to gathering information regarding applications of ChatGPT in health care, the survey focused on perceptions related to use in health care research, education, and practice. Participants reported uncertainty about the use of ChatGPT due to its recent release, although many respondents indicated an interest in future use. Similarly, in a survey of health care workers in Saudi Arabia, participants expressed concerns about ChatGPT use in health care, including lack of credibility and concerns about inaccurate medical information [8]. Despite these concerns, most respondents still anticipated a positive impact of ChatGPT on the future of health care practice, including medical decision-making, patient and family support, and medical research appraisal. Studies conducted in Jordan, Saudi Arabia, and the United Arab Emirates have reported similar findings among pharmacists [9-11].

These findings were reinforced in a 2023 systematic review that examined 60 studies focusing on the applications of and concerns about ChatGPT in health care practice, education, and research [6]. Potential uses for ChatGPT in practice included clinical documentation, creation of personalized care plans, improved health literacy, and provision of patient education. In education, ChatGPT was used in the creation of personalized learning tools and writing clinical cases. In research, ChatGPT was found to be beneficial in improving writing efficiency. However, in nearly all the studies, there were reported concerns with ChatGPT, including ethical, legal, and copyright issues; inaccuracy; and limited reproducibility. Specific to pharmacy, the use of ChatGPT has been explored with respect to drug information, medication therapy management, patient education, and adverse drug reaction assessment, with the general consensus that it has potential as a supplementary tool but is not yet able to handle complex problems [12-15].

On the basis of the findings from previously conducted surveys and research studies, it is evident that there is current and future potential for the use of AI technology in health care. However, information about the opinions and perceptions of pharmacists regarding the utility of AI chatbots in practice is limited. Pharmacists have been poorly represented in most surveys of health professionals conducted to date, and most studies regarding pharmacist perceptions have been conducted outside of the United States. Understanding the current perceptions, use, and barriers to use of chatbots will help inform and identify future roles for chatbots in pharmacy practice. Pharmacy preceptors are at the forefront of practice and education in the United States; therefore, this study aimed to assess current perceptions and use of AI chatbots in pharmacy practice from the perspective of pharmacist preceptors and determine factors that may influence the use of AI chatbots according to the Technology Acceptance Model Edited to Assess ChatGPT Adoption (TAME-ChatGPT) tool [16].

## Methods

### Overview

A cross-sectional survey was conducted using a convenience sample of pharmacy practice preceptors from Purdue University College of Pharmacy, University of Michigan College of Pharmacy, Wayne State University Eugene Applebaum College of Pharmacy and Health Sciences, University of Illinois Chicago Retzky College of Pharmacy, and Butler University College of Pharmacy and Health Sciences. Current pharmacist preceptors were recruited through their respective offices of experiential education. Pharmacists were excluded from the study if they had not precepted students in the previous year given the recent introduction of AI chatbots or if they did not practice in one of the affiliated states of Illinois, Indiana, or Michigan. Participants were instructed to only take the survey once.

### Questionnaire Design and Administration

The survey instrument was built in Qualtrics XM (Qualtrics International Inc) and was primarily based on the TAME-ChatGPT survey tool, a validated tool adapted from the technology acceptance model (TAM) for assessing health care students’ attitudes toward ChatGPT [16]. Before the first survey item, respondents were asked a screening question to ensure that they met the study inclusion criteria. The first section gathered information regarding pharmacists’ practice setting and current use of AI chatbots. The second section contained questions from the TAME-ChatGPT that were adapted to detect an association with different factors of the TAM (perceived ease of use, perceived risk, technology or social influence, anxiety, perceived usefulness, and behavior) and the use of AI chatbots. The survey concluded with questions regarding demographic information and tasks in which preceptors would recommend the use of AI. The instrument was pilot-tested by 3 faculty members to ensure adequate formatting, comprehension, clarity, and completeness of the survey. The final survey instrument is available in Multimedia Appendix 1.

An email invitation to complete the survey instrument was sent to 1877 pharmacy preceptors on February 12, 2024. The survey link was accessible until April 22, 2024, and 3 reminder emails were sent out before the survey closed. Only fully completed surveys were included in the final analysis.

### Data Analysis

Data were described using means and SDs for continuous variables and medians and frequencies for nominal variables. Univariable analysis was conducted using the student 2-tailed *t* test for continuous variables and chi-square test for nominal variables. Normality was assessed using visual inspection of *Q*-*Q* plots.

An exploratory factor analysis was conducted to assess construct validity of the TAME-ChatGPT instrument. First, correlation matrices, the Bartlett test, and the Kaiser-Meyer-Olkin measure of sampling adequacy were assessed to ensure that proceeding with factor analysis was appropriate. Multicollinearity was assessed by examining the determinant of the correlation in the correlation matrix with a <0.00001 cutoff. If multicollinearity was present, items with *r* values greater than 0.8 were considered for removal. The number of factors included was based on performing parallel analysis in which an eigenvalue cutoff was determined from randomly generated correlation matrices and then compared with eigenvalues from the data. Factors were retained if the dataset eigenvalue was greater than the corresponding random eigenvalue. Promax rotation was used to allow for correlation of factors. The Cronbach α was calculated to assess internal consistency.

Multivariable analysis was conducted using logistic regression to assess the association between preceptor characteristics and the components of the TAME-ChatGPT with the outcome of use of AI or future use of AI. Variables included the TAME-ChatGPT constructs and factors that significantly predicted current or future AI use. The number of variables included in the model was limited based on the number of participants who responded that they used AI. Goodness of fit was evaluated using the Hosmer-Lemeshow test (*P*>.05 indicating model fit) along with −2 log likelihood ratio, with smaller values indicating improved fit. As a secondary analysis, the model’s ability to discriminate between those who reported AI use and those who did not was evaluated using a receiver operating characteristic curve. The area under the curve was used as a summary measure of the model’s discrimination. SPSS (version 29; IBM Corp) was used to conduct the data analysis. A *P* value of <.05 was considered statistically significant.

### Ethical Considerations

This study was determined to be exempt research by the institutional review boards at all participating institutions—Butler University, Purdue University, University of Illinois Chicago, University of Michigan, and Wayne State University. The survey responses were anonymous and confidential, and all responses were stored without any identifiers. After survey completion, respondents were directed to an optional link to receive a US $10 Amazon gift card as compensation for participation.

## Results

### Overview

A total of 235 responses were received. Responses were excluded from the analysis if they were incomplete (n=10, 4.3%); if the respondent practiced outside of Indiana, Illinois, or Michigan (n=4, 1.7%); or if the respondent had not been a preceptor in the previous year (n=27, 11.5%). The final response number was 194, which represented an overall response rate of 10.3% (194/1877). Respondent demographics can be found in Table 1, and they were generally representative of pharmacy preceptors with the exception of years and area of practice based on data from the American Association of Colleges of Pharmacy 2024 preceptor survey [17]. Of the 194 responses that met the inclusion criteria, 59 (30.4%) indicated that the respondents had used an AI chatbot before. A total of 51.5% (100/194) of the respondents indicated that they would continue or plan to start using chatbots in the future.

Among those who had used AI chatbots before and those who had not, demographics were similar with respect to age, gender, and years of practice (Table 2). Most respondents (146/194, 75.3%) indicated that they were unlikely to make a patient care decision based on information from a chatbot. Of the respondents who had used AI chatbots (59/194, 30.4%), there was a substantially higher percentage who practiced in academia and a lower percentage who practiced in the community setting than among those who had not used AI chatbots. Additionally, respondents who had coworkers who used AI chatbots or an institutional AI policy were more likely to have used an AI chatbot themselves.

ChatGPT was the most frequently reported chatbot used (55/59, 93%), followed by Bing Chat (10/59, 17%) and Google Bard, now Gemini (7/59, 12%). The most common uses for AI chatbots in practice included summarizing information (31/59, 53%), letter of recommendation writing (20/59, 34%), and obtaining disease state information (14/59, 24%). Among respondents who had not used AI chatbots in practice before (135/194, 69.6%), 63% (85/135) selected not knowing how to use them effectively as the reason for disuse. Other common reasons for disuse included preference for other resources (80/135, 59.3%), lack of credibility or trust (63/135, 46.7%), and concerns of plagiarism (43/135, 31.9%).

All 194 respondents received the survey including questions about the respondents’ recommendations for use of AI chatbots by pharmacists and pharmacy students in practice. The top recommendations for use for pharmacists included administrative purposes (92/194, 47.4%), summarizing information (90/194, 46.4%), creating meeting agendas (74/194, 38.1%), letter of recommendation writing (64/194, 33%), and obtaining disease state information (63/194, 32.5%); 15.5% (30/194) of respondents indicated that they would not recommend that pharmacists use AI chatbots in practice. In comparison, the top uses recommended for students were summarizing information (61/194, 31.4%), creating meeting agendas (45/194, 23.2%), obtaining disease state information (42/194, 21.6%), administrative purposes (42/194, 21.6%), and conducting literature searches (38/194, 19.6%). In total, 40.2% (78/194) of the respondents indicated that they would not recommend that students use AI chatbots in practice.

**Table 1:** Demographics of pharmacy preceptor respondents.

		Survey respondents (n=194), n (%)	Respondents to the American Association of Colleges of Pharmacy survey [17] (n=4739), n (%)
**Gender**			
	Woman	142 (73.2)	2609 (56.6)
	Man	48 (24.7)	1665 (36.1)
	Nonbinary or third gender	1 (0.5)	NR^a^
	Preferred not to self-describe	3 (1.5)	327 (7.1)
Age (years), mean (SD)		38.7 (9.4)	NR
**Years of practice**			
	0-5	55 (28.4)	588 (12.6)
	6-10	51 (26.3)	951 (20.4)
	11-15	32 (16.5)	922 (19.8)
	>15	56 (28.9)	2097 (45.1)
**Area of practice**			
	Hospital	112 (57.7)	1521 (32.9)
	Community	29 (14.9)	1251 (27.1)
	Academia	16 (8.2)	172 (3.7)
	Ambulatory	16 (8.2)	717 (15.5)
	Drug information	8 (4.1)	NR
	Managed care	7 (3.6)	85 (1.8)
	Long-term care	3 (1.5)	NR
	Industry	1 (0.5)	88 (1.9)
	Other	14 (7.2)	604 (13.1)
	Specialty	6 (3.1)	NR
	Consulting	1 (0.5)	NR
	Infusion	1 (0.5)	NR
	Research	1 (0.5)	NR
	Medication safety	1 (0.5)	NR

^a^NR: not reported.

**Table 2:** Comparison of demographics between respondents who had and had not used artificial intelligence (AI) chatbots.

		Had used AI chatbots (n=59), n (%)	Had not used AI chatbots (n=135), n (%)	*P* value	
Age (years), mean (SD)		38.7 (9.1)	38.7 (9.5)	>.99	
**Gender**					.40
	Woman	39 (66.1)	103 (76.3)		
	Man	19 (32.2)	29 (21.5)		
	Nonbinary or third gender	0 (0.0)	1 (0.7)		
	Preferred not to self-describe	1 (1.7)	2 (1.5)		
**Years of practice**					.41
	0-5	15 (25.4)	40 (29.6)		
	6-10	16 (27.1)	62 (45.9)		
	11-15	11 (18.6)	21 (15.6)		
	>15	17 (28.8)	39 (28.9)		
**Area of practice**					.002
	Hospital	38 (64.4)	74 (54.8)		
	Community	4 (6.8)	25 (18.5)		
	Ambulatory	12 (20.3)	24 (17.8)		
	Academia	11 (18.6)	5 (3.7)		
	Specialty	0 (0.0)	6 (4.4)		
**Coworker use of AI chatbots**					<.001
	Yes	25 (42.4)	9 (6.7)		
	No	9 (15.3)	69 (51.1)		
	Unsure	25 (42.4)	57 (42.2)		
**AI policy at practice site**					.02
	Yes	11 (18.6)	8 (5.9)		
	No	30 (50.8)	82 (60.7)		
	Unsure	18 (30.5)	45 (33.3)		
“**How likely would you be to make a health care (ie, patient care or treatment) related recommendation based on the information an AI Chatbot (eg, ChatGPT) provides you?”**					.33
	Extremely unlikely	32 (54.2)	57 (42.2)		
	Somewhat unlikely	16 (27.1)	41 (30.4)		
	Neither likely nor unlikely	9 (15.3)	25 (18.5)		
	Somewhat likely	2 (3.4)	12 (8.9)		
	Extremely likely	0 (0.0)	0 (0.0)		
“**How likely would you be to make a policy related decision based on the information an AI Chatbot (eg, ChatGPT) provides you?”**					.25
	Extremely unlikely	17 (28.8)	49 (36.3)		
	Somewhat unlikely	15 (25.4)	37 (27.4)		
	Neither likely nor unlikely	16 (27.1)	34 (25.2)		
	Somewhat likely	11 (18.6)	12 (8.9)		
	Extremely likely	0 (0.0)	3 (2.2)		

### Factors Associated With AI Use

The first 13 items of the TAME-ChatGPT were included in the primary exploratory factor analysis. These items included all survey respondents, with an overall rate of AI use of 30.4% (59/194). The overall data were appropriate for conducting factor analysis (Bartlett test χ^2^_78_=1288.7; *P*<.001), and the Kaiser-Meyer-Olkin value (0.82) indicated that sampling was adequate. There was no concern for multicollinearity, and parallel analysis identified 2 factors as the optimal number, with an eigenvalue cutoff of 1.31. The eigenvalues for the 2 factors were 4.49 and 1.97, which explained 49.7% of the cumulative variance. These 2 factors were classified as attitude toward the technology and perceived risk. Descriptive statistics for these constructs are provided in Table 3, and the pattern matrix is shown in Table 4. Including a third factor (eigenvalue=1.155), similar to the original TAME-ChatGPT validation study, resulted in an explanation of 57.1% of the cumulative variance. However, factors 2 and 3 were largely correlated (*r*=0.57), and many of the items were correlated with both factors 2 and 3. Cronbach α values for the 2 constructs were good at 0.86 for both comfort with technology and perceived risk.

In the secondary exploratory analysis, all items were included for the 30.4% (59/194) of respondents who had previously used AI. The overall data were appropriate for conducting factor analysis (Bartlett test χ^2^_528_=1301.5; *P*<.001), and the Kaiser-Meyer-Olkin value (0.70) indicated that sampling was adequate. There was concern for multicollinearity, and therefore, items 4, 14, and 31 were removed. Parallel analysis identified 4 factors as the optimal number, with an eigenvalue cutoff of 2.08. The eigenvalues for the 4 factors were 9.33, 4.36, 2.96, and 2.26, which explained 57.3% of the cumulative variance. The 4 factors identified included attitude toward the technology, perceived usefulness, perceived risk, and ease of use. Descriptive statistics for these constructs are provided in Table 3, and the pattern matrix is shown in Table 5. Cronbach α values for the 4 constructs were very reliable for attitude toward the technology (0.92), perceived usefulness (0.92), perceived risk (0.84), and ease of use (0.83).

**Table 3:** Technology Acceptance Model Edited to Assess ChatGPT Adoption constructs.

		Score, mean (SD)	*P* value	
**Perceived risk^a^**				<.001
	Had used AI^b^ chatbots before	27.92 (6.32)		
	Had not used AI chatbots before	30.59 (6.01)		
**Attitude toward technology^c^**				<.001
	Had used AI chatbots before	19.19 (3.63)		
	Had not used AI chatbots before	15.07 (4.06)		
Attitude toward technology among users^d^		44.53 (10.02)	—^e^	
Perceived usefulness among users^f^		14.08 (5.80)	—	
Perceived ease of use among users^g^		11.29 (2.54)	—	
Perceived risk of use among users^h^		33.75 (7.98)	—	

^a^Possible range from 8 to 40, with higher scores indicating lower perceived risk and a score of 24 indicating a neutral attitude.

^b^AI: artificial intelligence.

^c^Possible range from 5 to 25, with higher scores indicating positive attitude and a score of 15 indicating a neutral attitude.

^d^Possible range from 13 to 65, with higher scores indicating positive attitude and a score of 39 indicating a neutral attitude.

^e^Not applicable.

^f^Possible range from 6 to 30, with higher scores indicating higher perceived usefulness and a score of 18 indicating a neutral attitude.

^g^Possible range from 3 to 15, with higher scores indicating perceived ease of use and a score of 9 indicating a neutral attitude.

^h^Possible range from 5 to 50, with higher scores indicating lower perceived risk and a score of 27.5 indicating a neutral attitude.

**Table 4:** Pattern matrix of the 2 inferred factors for all respondents irrespective of previous artificial intelligence (AI) chatbot use.

Item	Perceived risk	Attitude toward technology
“I am concerned about the reliability of the information provided by AI chatbots.”	0.435	<0.400
“I am concerned that using AI chatbots is considered plagiarism.”	0.617	<0.400
“I fear relying too much on AI chatbots may decrease my critical thinking skills.”	0.585	<0.400
“I am concerned about the potential security risks of using AI chatbots.”	0.798	<0.400
“I am afraid of becoming too dependent on technology like AI chatbots.”	0.618	<0.400
“I am afraid that using AI chatbots would result in a lack of originality in my work.”	0.688	<0.400
“I am afraid that the use of the AI chatbots would be a violation of workplace policies.”	0.655	<0.400
“I am concerned about the potential privacy risks that might be associated with using AI chatbots.”	0.786	<0.400
“I am enthusiastic about using technology, such as AI chatbots for learning, practice, and research.”	<0.400	0.887
“I believe technology, such as AI chatbots is an important tool for workplace success.”	<0.400	0.844
“I think that technology like AI chatbots is attractive and fun to use.”	<0.400	0.871
“I am always open to learning about new technologies like AI chatbots.”	<0.400	0.672
“I trust the opinions of my friends or colleagues about using AI chatbots.”	<0.400	0.444

Logistic regression was conducted to predict current and future use of AI (Table 6). Factors that remained significant in the model that predicted current AI use included positive attitude toward technology, coworker use of AI, and the respondent practicing in academia. Factors that predicted future use of AI included perceived risk, positive attitude toward technology, and coworker use.

**Table 5:** Pattern of the 4 inferred factors for only those who had used artificial intelligence (AI) chatbots before.

Item	Attitude toward technology	Perceived usefulness	Ease of use	Perceived risk
“I am concerned about the reliability of the information provided by AI chatbots.”	<0.400	<0.400	<0.400	0.530
“I am concerned that using AI chatbots is considered plagiarism.”	<0.400	<0.400	<0.400	0.652
“I fear relying too much on AI chatbots may decrease my critical thinking skills.”	<0.400	<0.400	<0.400	0.457
“I am afraid of becoming too dependent on technology like AI chatbots.”	<0.400	<0.400	<0.400	0.746
“I am afraid that using AI chatbots would result in a lack of originality in my work.”	<0.400	<0.400	<0.400	0.762
“I am afraid that the use of the AI chatbots would be a violation of workplace policies.”	<0.400	<0.400	<0.400	0.582
“I am concerned about the potential privacy risks that might be associated with using AI chatbots.”	<0.400	<0.400	<0.400	0.750
“I am enthusiastic about using technology, such as AI chatbots for learning, practice, and research.”	0.514	<0.400	<0.400	<0.400
“I believe technology, such as AI chatbots is an important tool for workplace success.”	0.611	<0.400	<0.400	<0.400
“I think that technology like AI chatbots is attractive and fun to use.”	0.609	<0.400	<0.400	<0.400
“I am always open to learning about new technologies like AI chatbots.”	0.498	<0.400	<0.400	<0.400
“I trust the opinions of my friends or colleagues about using AI chatbots.”	<0.400	<0.400	<0.400	−0.571
“For me, AI chatbots are a convenient method for accessing medical information.”	<0.400	0.913	<0.400	<0.400
“For me, AI chatbots are a reliable source of accurate medical information.”	<0.400	0.855	<0.400	<0.400
“AI chatbots help me in better understanding of difficult medical topics and concepts.”	<0.400	0.878	<0.400	<0.400
“AI chatbots make it easier for me to complete tasks in my workplace.”	0.784	<0.400	<0.400	<0.400
“I recommend AI chatbots to my colleagues to facilitate their work.”	0.807	<0.400	<0.400	<0.400
“AI chatbots are more useful than other sources of medical information that I have used previously.”	<0.400	0.843	<0.400	<0.400
“I think that using AI chatbots has helped to improve my overall workplace performance.”	0.737	<0.400	<0.400	<0.400
“I have used tools similar to AI chatbots in the past in my workplace.”	0.679	<0.400	<0.400	<0.400
“I spontaneously find myself using AI chatbots when I need medical information for my work.”	<0.400	0.731	<0.400	<0.400
“I often use AI chatbots as a source of medical information in my workplace.”	<0.400	0.766	<0.400	<0.400
“I appreciate the convenience and efficiency that AI chatbots provide for my work.”	0.690	<0.400	<0.400	<0.400
“I think that relying on technology like AI chatbots can disrupt my critical thinking skills.”	<0.400	<0.400	<0.400	0.674
“I appreciate the accuracy and reliability of the medical information provided by AI chatbots.”	<0.400	0.821	<0.400	<0.400
“I believe that using AI chatbots can save time and effort in my workplace.”	0.828	<0.400	<0.400	<0.400
“It does not take a long time to learn how to use AI chatbots.”	<0.400	<0.400	0.782	<0.400
“Using AI chatbots does not require extensive technical knowledge.”	<0.400	<0.400	0.828	<0.400
“I do not face many difficulties when using AI chatbots.”	<0.400	<0.400	0.787	<0.400
“The positive experiences of others have encouraged me to use AI chatbots.”	0.486	<0.400	<0.400	<0.400
“I believe that people I know have improved their workplace performance as a result of using AI chatbots.”	0.796	<0.400	<0.400	<0.400
“I think using AI chatbots is important for me to keep up with my peers professionally.”	0.542	<0.400	<0.400	<0.400

**Table 6:** Predictors of current and future artificial intelligence (AI) chatbot use.

			OR^a^ (95% CI)		*P* value
**Current use of AI^b^**					
	Perceived risk	0.98 (0.61-1.56)		.94	
	Attitude toward technology	3.64 (2.08-6.36)		<.001	
	Coworker use of AI	7.41 (2.64-20.80)		<.001	
	AI policy present	2.72 (0.80-9.20)		.11	
	Academia	5.62 (1.30-24.23)		.02	
	Community	0.52 (0.14-1.90)		.32	
**Future use of AI^c^**					
	Perceived risk	0.63 (0.41-0.96)		.03	
	Attitude toward technology	4.11 (2.42-6.97)		<.001	
	Coworker use of AI	33.00 (5.02-216.76)		<.001	
	AI policy present	2.24 (0.60-8.43)		.23	
	Academia	2.06 (0.42-10.21)		.38	
	Community	1.09 (0.41-2.89)		.86	

^a^OR: odds ratio.

^b^Hosmer and Lemeshow *P*=.15; area under the curve 0.85.

^c^Hosmer and Lemeshow *P*=.71; area under the curve 0.87.

## Discussion

Approximately one-third of pharmacy preceptors (59/194, 30.4%) reported use of an AI chatbot, with approximately half (100/194, 51.5%) indicating that they planned to start or would continue using chatbots in the future. Consistent with findings from other studies, we found that most respondents were unlikely to make patient care decisions based on information provided by an AI chatbot. However, they did report use for administrative tasks such as summarizing information and writing letters of recommendation. To our knowledge, this is the first study using the TAME-ChatGPT assessment tool among pharmacists. The findings show that this tool is valid and reliable for assessing pharmacists’ attitudes toward chatbots and their use in pharmacy practice. Pharmacists’ attitudes toward chatbots were largely influenced by their attitude toward the technology and their perceived risk related to use of the technology. Furthermore, among pharmacists who had used chatbots, attitudes toward use of chatbots were affected by the same 2 factors plus their perceived usefulness and ease of use. Positive attitudes toward technology, having coworkers who use AI, and working in academia predicted current use of AI chatbots, whereas factors predicting future use of the technology included perceived risk, positive attitudes toward technology, and coworker use of AI chatbots. Our results affirm that, when adopting ChatGPT and other AI chatbots, it is important to consider perceptions of risk, usefulness, and ease of use, as well as the users’ attitudes toward technology.

Significantly more pharmacists practicing in academia have used chatbots than pharmacists in other practice settings, perhaps because these pharmacists are often at the forefront of exploring new technologies and their applications in pharmacy practice and may be more comfortable with adopting new technologies. While academic pharmacists may be at the forefront of chatbot adoption, the use of AI chatbots is expected to increase across all pharmacy practice settings as the technology becomes more refined and its benefits become more apparent. Since the time of our data collection, more advanced AI chatbots have become available, such as OpenEvidence, which may provide more targeted information for pharmacists to use in practice.

However, most pharmacists surveyed (135/194, 69.6%) had not used an AI chatbot, with nearly two-thirds of them (85/135, 63%) stating that their reason for not using chatbots was that they did not know how to use them effectively; 46.7% (63/135) cited lack of credibility or trust in chatbots as the reason for lack of use. AI chatbots are relatively new technologies in health care, and many pharmacists may not have had sufficient exposure or training to use them effectively. Furthermore, there is currently no standardized approach to training pharmacists or other health care providers on the use of AI chatbots; thus, knowledge and skills across the profession may be inconsistent. Knowledge on how to use AI chatbots will likely improve as they become more prevalent in health care settings. Pharmacists will gain hands-on experience with these tools, become more comfortable using them, and learn to integrate them into their daily workflows. Preceptors are in an ideal position to help guide students who are just learning about AI capabilities and limitations on the optimal use of this new technology. Health care organizations, educational institutions, and postgraduate training programs should consider including training on the appropriate use of AI and risks of inappropriate use.

Our findings differ somewhat from those of the work by Sallam et al [16], who validated the TAME-ChatGPT in a sample of Jordanian health care students and identified factors affecting their attitudes toward ChatGPT and use of ChatGPT. Attitudes of Jordanian students toward ChatGPT were influenced by an additional third factor, anxiety related to fear of ChatGPT; attitudes toward use of ChatGPT were affected by behavior as a fourth factor instead of attitudes toward technology [16]. The differences are likely due to differences in the populations studied; US pharmacists and Jordanian students represent very different populations based on culture, age, practice experience, and other characteristics. Different approaches to factor analysis may also contribute to differences.

Risk perception is known to be a key factor affecting decision-making, and perception of risks associated with chatbots significantly influenced pharmacists’ attitudes toward chatbots and their use [18]. The credibility of AI chatbots in health care is a complex and evolving issue. Reliability and accuracy can vary significantly between chatbots, and not all AI chatbots are reliable sources of information. Inaccuracies, hallucinations, potential for biased responses, and the inability of chatbots to provide nuanced or context-specific information have been documented [12,19-21]. These potential barriers highlight the need for health care professional scrutiny and oversight of chatbot responses. As the technologies continue to develop, ongoing research and validation will be crucial to establish and maintain the credibility of AI chatbots in health care applications and minimize their potential for harm.

Some of the limitations of this study include the sample size as only pharmacist preceptors in the Midwest who were affiliated with the participating colleges were surveyed, so the results may not be representative of pharmacy preceptors across the United States. However, based on comparison with the 2024 American Association of Colleges of Pharmacy preceptor survey, outside of years and area of practice, the surveyed sample seems to be fairly representative of preceptors in the United States [17]. Our response rate was low, which may be due to technology-related factors (ie, the email going to the spam folder) and may have resulted in selection bias in the sense that preceptors with experience using AI may have been more likely to respond. As the survey was anonymous, we could not explore differences between respondents and nonrespondents. It is possible that the use of AI at the time of our survey overestimated use in the target population. Additionally, the AI and chatbot space is constantly evolving, and although the survey was conducted recently, perceptions can change rapidly as more people are exposed to chatbots and start using them in their daily work. Future studies could expand the surveyed population to include pharmacist preceptors nationwide or include all pharmacists, along with following up on respondents over time to evaluate changes in responses. Additionally, the validated survey can be readministered following training and education on AI chatbots to determine how perceptions of AI technology have changed.

At the time of our study, it had been almost 2 years since AI chatbots were introduced, and pharmacist preceptors were still hesitant to use the new technology, with only approximately one-third of respondents (59/194, 30.4%) indicating that they had used a chatbot in practice. Pharmacist preceptors were hesitant to use the technology for clinical decisions and were uncertain about their place in practice. This study also demonstrated that the TAME-ChatGPT survey is a reliable and validated tool that can be used to assess pharmacists’ attitude toward and use of chatbots. Constructs from the TAM, including attitude toward technology, perceived usefulness, ease of use, and perceived risk of use, as well as practice setting and coworker use, can determine and predict pharmacist use of AI chatbots. Future studies with this validated tool can be used to guide the implementation of chatbots into pharmacy practice and help inform policymakers and organization leaders on the education and training needed to promote the safe and effective use of AI chatbots in pharmacy practice.

## Appendix

Multimedia Appendix 1 Survey instrument.

## Abbreviations

AI: artificial intelligence
OR: odds ratio
TAM: technology acceptance model
TAME-ChatGPT: Technology Acceptance Model Edited to Assess ChatGPT Adoption
