# Supplementary material for: Assessing Pharmacists’ Use and Perception of AI Chatbots in Pharmacy Practice: Cross-Sectional Survey Study
Source: JMIR Med Educ. 2025 Nov 21;11:e71767. doi: 10.2196/71767 (PMC12680932; doi:10.2196/71767)
Supplement: Multimedia Appendix 1 [file mededu_v11i1e71767_app1.pdf]

- You are at least 18 years old

○ I agree
○ I do NOT agree

Have you served as a preceptor for a pharmacy student in the past year?

○ Yes
○ No

In what state do you currently practice?

○ Illinois
○ Indiana
○ Michigan
○ [ ] Other (please specify):

What is your **primary** area of practice (select all that apply)?

☐ Academia
☐ Ambulatory
☐ Community

- ☐ Drug information
- ☐ Hospital
- ☐ Industry
- ☐ Long-term care
- ☐ Managed care
- ☐ [ ] Other (please specify):

How many years have you been in practice (round to nearest whole number)?

[ ]

AI chatbots include tools like ChatGPT, GoogleBard, BingChat, and DougallGPT. Which of the following best describes how you use AI chatbots in practice?

- ○ I have never used them and I do not plan to use them
- ○ I have never used them, but I plan to use them
- ○ I have used them before but do not plan to use them moving forward
- ○ I have used them before and plan to continue using them

Which AI chatbots have you used before in practice (select

all that apply)?

- [ ] ChatGPT
- [ ] Google Bard
- [ ] Bing Chat
- [ ] DougallGPT
- [ ] Perplexity AI
- [ ] YouChat
- [ ] LLaMA
- [ ] Other (please specify)

Do your coworkers use AI chatbots in practice?

- ○ Yes
- ○ No
- ○ Unsure

Does your practice setting have a policy for use of AI chatbots?

- ○ Yes
- ○ No
- ○ Unsure

Which of the following best describes the policy for AI chatbots in your practice setting?

- ○ Does not allow use
- ○ Allows use with restrictions
- ○ Allows use without restrictions

Why have you not used AI chatbots in practice before (select all that apply)?

- ☐ Never heard of them
- ☐ Not interested in using them
- ☐ Not sure how to use them effectively
- ☐ Lack of credibility/do not trust them
- ☐ Concerns of plagiarism
- ☐ Prefer to use other resources (Guidelines, UpToDate, Lexicomp, primary literature)
- ☐ Institutional policies prohibit use/are unclear
- ☐ Lack of access
- ☐ Concerns about privacy/did not want to register for an account
- ☐ Other (please specify): ____________

If you have used AI chatbots in practice, what did you use them for (select all that apply)?

- [ ] Obtaining drug information
- [ ] Administrative purposes (e.g. scheduling, charting, data entry)
- [ ] Obtaining disease state information
- [ ] Patient education
- [ ] Evaluating patient information
- [ ] Summarizing information
- [ ] Conducting literature searches
- [ ] Policy/procedure creation
- [ ] Grading
- [ ] Letter of recommendation writing
- [ ] Medical/scholarly writing
- [ ] Creating meeting agendas
- [ ] Research (e.g., formulating research questions)
- [ ] Clinical documentation
- [ ] Clinical decision-making support
- [ ] Drafting exam questions
- [ ] Quality improvement
- [ ] Other (please specify ______

How likely would you be to make a healthcare (i.e., patient care or treatment) related recommendation based on the information an AI Chatbot (e.g., Chat GPT) provides you?

- ○ Extremely likely
- ○ Somewhat likely
- ○ Neither likely nor unlikely
- ○ Somewhat unlikely
- ○ Extremely unlikely

How likely would you be to make a policy related decision based on the information an AI Chatbot (e.g., Chat GPT) provides you?

- Extremely likely
- Somewhat likely
- Neither likely nor unlikely
- Somewhat unlikely
- Extremely unlikely

Has not used chatbots

Please indicate your level of agreement with the following statements on a scale of 1 to 5, where 1 is strongly disagree, and 5 is strongly agree.

	1<br>Strongly disagree	2<br>Somewhat disagree	3<br>Neither agree nor disagree	4<br>Somewhat agree	5<br>Strongly agree
I am concerned about the reliability of the information provided by AI chatbots	○	○	○	○	○
I am concerned that using AI chatbots is considered plagiarism	○	○	○	○	○

	1	2	3	4	5
	Strongly disagree	Somewhat disagree	Neither agree nor disagree	Somewhat agree	Strongly agree
I fear relying too much on AI chatbots may decrease my critical thinking skills	○	○	○	○	○
I am concerned about the potential security risks of using AI chatbots	○	○	○	○	○
	Strongly disagree	Somewhat disagree	Neither agree nor disagree	Somewhat agree	Strongly agree
I am afraid of becoming too dependent on technology like AI chatbots	○	○	○	○	○
I am afraid that using AI chatbots would result in a lack of originality in my work	○	○	○	○	○
I am afraid that the use of the AI chatbots would be a violation of workplace policies	○	○	○	○	○
I am concerned about the potential privacy risks that might be associated with using AI chatbots	○	○	○	○	○

Please indicate your level of agreement with the following statements on a scale of 1 to 5, where 1 is strongly disagree, and 5 is strongly agree.

	1<br>Strongly disagree	2<br>Somewhat disagree	3<br>Neither agree nor disagree	4<br>Somewhat agree	5<br>Strongly agree
I am enthusiastic about using technology such as AI chatbots for learning, practice, and research	○	○	○	○	○
I believe technology such as AI chatbots is an important tool for workplace success	○	○	○	○	○
I think that technology like AI chatbots is attractive and fun to use	○	○	○	○	○
I am always open to learning about new technologies like AI chatbots	○	○	○	○	○
I trust the opinions of my friends or colleagues about using AI chatbots	○	○	○	○	○

Has used chatbots

Please indicate your level of agreement with the following statements on a scale of 1 to 5, where 1 is strongly disagree, and 5 is strongly agree.

	1<br>Strongly disagree	2<br>Somewhat disagree	3<br>Neither agree nor disagree	4<br>Somewhat agree	5<br>Strongly agree
AI chatbots help me to save time when searching for medical information	○	○	○	○	○
For me, AI chatbots are a convenient method for accessing medical information	○	○	○	○	○
For me, AI chatbots are a reliable source of accurate medical information	○	○	○	○	○
AI chatbots help me in better understanding of difficult medical topics and concepts	○	○	○	○	○
	Strongly disagree	Somewhat disagree	Neither agree nor disagree	Somewhat agree	Strongly agree
AI chatbots make it easier for me to complete tasks in my workplace	○	○	○	○	○
I recommend AI chatbots to my colleagues to facilitate their work	○	○	○	○	○

	1<br>Strongly disagree	2<br>Somewhat disagree	3<br>Neither agree nor disagree	4<br>Somewhat agree	5<br>Strongly agree
AI chatbots are more useful than other sources of medical information that I have used previously	○	○	○	○	○
I think that using AI chatbots has helped to improve my overall workplace performance	○	○	○	○	○

	Strongly disagree	Somewhat disagree	Neither agree nor disagree	Somewhat agree	Strongly agree
I have used tools similar to AI chatbots in the past in my workplace	○	○	○	○	○
I spontaneously find myself using AI chatbots when I need medical information for my work	○	○	○	○	○
I often use AI chatbots as a source of medical information in my workplace	○	○	○	○	○
I appreciate the convenience and efficiency that AI chatbots provide for my work	○	○	○	○	○

Please indicate your level of agreement with the following statements on a scale of 1 to 5, where 1 is strongly disagree, and 5 is strongly agree.

	1<br>Strongly disagree	2<br>Somewhat disagree	3<br>Neither agree nor disagree	4<br>Somewhat agree	5<br>Strongly agree
I think that relying on technology like AI chatbots can disrupt my critical thinking skills	○	○	○	○	○
I appreciate the accuracy and reliability of the medical information provided by AI chatbots	○	○	○	○	○
I believe that using AI chatbots can save time and effort in my workplace	○	○	○	○	○
I appreciate the importance of using traditional resources to find medical information, even if it means not relying on technology like AI chatbots	○	○	○	○	○
	Strongly disagree	Somewhat disagree	Neither agree nor disagree	Somewhat agree	Strongly agree
It does not take a long time to learn how to use AI chatbots	○	○	○	○	○

	1	2	3	4	5
	Strongly disagree	Somewhat disagree	Neither agree nor disagree	Somewhat agree	Strongly agree
AI chatbots are easy to use	○	○	○	○	○
Using AI chatbots does not require extensive technical knowledge	○	○	○	○	○
I do not face many difficulties when using AI chatbots	○	○	○	○	○
	Strongly disagree	Somewhat disagree	Neither agree nor disagree	Somewhat agree	Strongly agree
The positive experiences of others have encouraged me to use AI chatbots	○	○	○	○	○
I believe that people I know have improved their workplace performance as a result of using AI chatbots	○	○	○	○	○
I think using AI chatbots is important for me to keep up with my peers professionally	○	○	○	○	○

Recommend use AI chatbots

What would you recommend pharmacists use AI chatbots for in practice?

- ☐ Obtaining drug information
- ☐ Obtaining disease state information
- ☐ Evaluating patient information
- ☐ Conducting literature searches
- ☐ Grading
- ☐ Clinical decision-making support
- ☐ Medical/scholarly writing
- ☐ Quality improvement
- ☐ Research (e.g., formulating research questions)
- ☐ Patient education
- ☐ Administrative purposes (e.g. scheduling, charting, data entry)
- ☐ Summarizing information
- ☐ Policy/procedure creation
- ☐ Letter of recommendation writing
- ☐ Creating meeting agendas
- ☐ Clinical documentation
- ☐ Drafting exam questions
- ☐ Other (please specify ______
- ☐ I would not recommend pharmacists use AI chatbots

What would you recommend students use AI chatbots for in practice (on their IPPE/APPEs)?

- ☐ Obtaining drug information
- ☐ Obtaining disease state information
- ☐ Evaluating patient information
- ☐ Conducting literature searches
- ☐ Grading
- ☐ Administrative purposes (e.g. scheduling, charting, data entry)
- ☐ Summarizing information
- ☐ Policy/procedure creation
- ☐ Letter of recommendation writing
- ☐ Creating meeting agendas

- ☐ Clinical decision-making support
- ☐ Medical/scholarly writing
- ☐ Quality improvement
- ☐ Research (e.g., formulating research questions)
- ☐ Patient education
- ☐ Clinical documentation
- ☐ Drafting exam questions
- ☐ Other (please specify
- ☐ I would not recommend students use AI chatbots

Demographics

What is your age:

How would you describe your gender?

- ○ Woman
- ○ Man
- ○ Non-binary / third gender
- ○ Prefer not to self-describe
